# S6-3 Barriers, facilitators and strategies to implement physical activity advice and referral to exercise therapy for people with hip and knee OA in primary care

**DOI:** 10.1093/eurpub/ckad133.030

**Published:** 2023-09-11

**Authors:** Dieuwke Schiphof, Jurgen Damen, Sita Bierma-Zeinstra, Marienke van Middelkoop

**Affiliations:** Erasmus MC University Medical Center; Erasmus MC University Medical Center; Erasmus MC University Medical Center; Erasmus MC University Medical Center

## Abstract

**Aim:**

Osteoarthritis (OA) is one of the most common diseases in the Netherlands. Most people with OA are seen in primary care. Core treatment for knee and hip OA in general practice is information and advice on physical activity and referral to exercise therapy. All Dutch guidelines on osteoarthritis recommend exercise therapy before a referral to secondary care. Despite the guidelines, only around 60% of the people on the waiting list for a replacement surgery have received exercise therapy. This study aims to provide insight in barriers and facilitators of General Practitioners (GPs) and possible strategies for advice on physical activity and referral to exercise therapy for people with knee and hip OA.

**Method:**

A literature searched was conducted to identify barriers and facilitators of healthcare professionals (HCPs) to give people with OA advice about being physically active and refer them to exercise therapy. In two focus groups with GPS the identified barriers and facilitators were discussed and prioritized. A third focus group was used to prioritize the barriers and facilitators. The prioritized barriers and facilitators were classified within the Consolidated Framework for Implementation Research (CFIR). In a third meeting the prioritizing was checked and the first ideas for strategies, based on the Expert Recommendations for Implementing Change (ERIC) tool, were discussed.

**Results:**

Barriers and facilitators out of seven papers were discussed. Most important barriers found in two focus groups (N = 12) were: time, lack of uniform information of HCPs, and cooperation between different HCPs. In the third meeting with other GPs (N = 32) different barriers in addition to the existing barriers, were mentioned: doubts about effectivity and patients’ expectations. Ideas about strategies based on the ERIC tool were education, involvement of patients, organizing regional meetings with different HCPs.

**Conclusion:**

Different barriers were mentioned as most important in the focus groups, but all GPs agreed on the importance of the barrier of cooperation with different HCPs and uniform information to people with OA. Strategies that will be further developed, tested and evaluated are education for HCPs, organizations of interprofessional regional meetings and development of uniform information for people with OA.

